# Supplementary material for: Association of Life’s Simple 7 lifestyle metric with cardiometabolic disease-free life expectancy in older British men
Source: Commun Med (Lond). 2024 Jun 4;4:104. doi: 10.1038/s43856-024-00534-7 (PMC11150484; doi:10.1038/s43856-024-00534-7)
Supplement: Supplementary file 2 — Supplementary Information [file 43856_2024_534_MOESM2_ESM.pdf]

## Supplementary Information

**Supplementary Table 1.**
Life's Simple 7 metric definitions of poor, intermediate, and ideal for men participating in the British Regional Heart Study.

| LS7 Metric | Poor | Intermediate | Ideal |
|---|---|---|---|
| BMI, kg/m$^2$ | ≥ 30 | 25-30 | < 25 |
| Blood pressure, mmHg | SBP≥140 or DBP≥90 | SBP 120-140 or DBP 80-90 or treated to goal | SBP < 120, DBP < 80 and untreated |
| Blood glucose [a], mmol/L | N/A | 5.6-7.0 or treated to goal | < 5.6 and untreated |
| Total cholesterol, mmol/L | ≥ 6.2 | 5.2-6.2 | < 5.2 |
| Smoking | Current smoking | Gave up ≤ 5 years | Never or gave up > 5 years |
| Physical activity [b] | Inactive | Occasional to light activity | Moderate to vigorous activity |
| Diet [c] | EDI score 12-23 | EDI score 24-26 | EDI score 27-35 |

[a] Blood glucose ≥ 7.0 mmol/L was defined as screen-detected type 2 diabetes.
[b] Physical activity level was derived based on the frequency and type (intensity) of self-reported usual pattern of physical activity, including regular walking or cycling, recreational, and sporting (vigorous) activity. In specific, based on the frequency and type (intensity), the self-reported physical activity information was firstly summarized into a score based on the recommendations of a National Heart, lung, and Blood Institute workshop and the Minnesota intensity codes[30,31], and then the scores were grouped into six broad categories: 0-2 inactive; 3-5 occasional activity; 6-8 light activity; 9-12 moderate activity; 13-20 moderately vigorous activity; and ≥ 21 vigorous activity. The LS7 physical activity metric was created by further categorizing inactive as poor; occasional to light as intermediate; and moderate to vigorous activity as ideal.
[c] Diet was assessed by the Elderly Dietary Index (EDI) score, which is a dietary score that developed specifically to address older adults' adherence to the nutritional recommendations[27]. The EDI consists of 9 components (fruit, vegetables, meat, fish/seafood, cereals, legumes, bread, olive oil, and dairy). Each component was assigned a 4-point scoring system based on the consumption frequency (1, 2, 3, 4, 5, 6, or 7 days/wk; monthly; or rarely/never). This resulted in a total EDI score range from 9 to 36, with higher scores indicating a presumably healthier diet. The LS7 diet metric was derived by categorizing participants into tertiles of EDI scores: 12-23 (poor); 24-26 (intermediate); and 27-35 (ideal).
BMI = body mass index; DBP = diastolic blood pressure; EDI = elderly diet index; SBP = systolic blood pressure.

**Supplementary Table 2.**
Comparison of complete cases and missing sample, among BRHS participants free of prevalence cardiometabolic diseases at Q20 (n=3167).

| Variable | Complete cases (n=2662) | Missing sample (n=505) | p-value |
|---|---|---|---|
| CMD/Death events (%) | 1764 (66.3) | 390 (77.2) | |
| Incidence / 1000PY | 33.1 | 38.6 | |
| Age at baseline, years | 68.2 (5.5) | 69.7 (5.8), N = 505 | < 0.01 |
| Social Class | | N = 505 | < 0.01 |
| Manual | 45.7 | 51.3 | |
| Non-manual | 50.6 | 41.2 | |
| Unspecified | 3.8 | 7.5 | |

| | | | |
|---|---|---|---|
| Alcohol Intake | | N = 505 | < 0.01 |
| None | 8.7 | 13.9 | |
| Occasional | 25.9 | 24.4 | |
| Light | 44.9 | 40.8 | |
| Moderate | 16.2 | 12.3 | |
| Heavy | 2.8 | 4.2 | |
| Unspecified | 1.5 | 4.6 | |
| Energy Intake | 2152.8 (519.0) | 2059.9 (575.5), N = 414 | < 0.01 |
| BMI | | N = 496 | < 0.01 |
| Poor | 13.7 | 20.6 | |
| Intermediate | 53.2 | 51.2 | |
| Ideal | 33.1 | 28.2 | |
| Blood Pressure | | N = 473 | 0.63 |
| Poor | 40.8 | 40.4 | |
| Intermediate | 51.6 | 50.7 | |
| Ideal | 7.6 | 8.9 | |
| Blood Glucose | | N = 354 | 0.84 |
| Intermediate | 46.6 | 47.2 | |
| Ideal | 53.4 | 52.8 | |
| Total Cholesterol | | N = 356 | 0.07 |
| Poor | 45.8 | 39.9 | |
| Intermediate | 36.3 | 38.5 | |
| Ideal | 17.8 | 21.6 | |
| Smoking | | N = 499 | 0.61 |
| Poor | 13.0 | 14.6 | |
| Intermediate | 2.6 | 2.6 | |
| Ideal | 84.4 | 82.8 | |
| Physical Activity | | N = 401 | < 0.01 |
| Poor | 8.7 | 12.2 | |
| Intermediate | 41.0 | 47.4 | |
| Ideal | 50.3 | 40.4 | |
| Diet | | N = 273 | 0.06 |
| Poor | 38.7 | 45.8 | |
| Intermediate | 37.2 | 34.8 | |
| Ideal | 24.0 | 19.4 | |

Values are presented as Mean (SD) or percentage unless stated otherwise.
Pearson Chi-squared test was used for all categorical variables.
Wilcoxon rank sum test was used for all continuous variables.
BMI = body mass index; CMD = cardiometabolic disease; LS7 = Life's Simple 7.

**Supplementary Table 3.**
The Hazard Ratios (HRs) of the associations between Life's Simple 7 adherence and risk of cardiometabolic diseases/death in BRHS participants aged 60-79 in 1998-2000, after imputing missing covariates (n=3167).

| LS7 Adherence | HR | 95% CI | p-value |
|---|---|---|---|
| Poor<br>(Achieved 0-1 LS7 ideal levels) | Ref | - | < 0.01 |
| Intermediate<br>(Achieved 2-3 LS7 ideal levels) | 0.72 | (0.64, 0.81) | |
| Ideal<br>(Achieved 4+ LS7 ideal levels) | 0.57 | (0.49, 0.66) | |

Models used age as time scale, and adjusted for social class, alcohol intake, and energy intake.
LS7 = Life's Simple 7.

**Supplementary Table 4.**
The Hazard Ratios (HRs) of the associations between Life's Simple 7 adherence and risk of cardiometabolic diseases/death in BRHS participants aged 60-79 in 1998-2000, further adjusted for IMD (n=2662).

| LS7 Adherence | HR | 95% CI | p-value |
|---|---|---|---|
| Poor (Achieved 0-1 LS7 ideal levels) | Ref | - | < 0.01 |
| Intermediate (Achieved 2-3 LS7 ideal levels) | 0.74 | (0.65, 0.84) | |
| Ideal (Achieved 4+ LS7 ideal levels) | 0.59 | (0.51, 0.70) | |

IMD: National index of Multiple Deprivation (IMD).
Models used age as time scale, and adjusted for social class, alcohol intake, energy intake, and IMD. LS7 = Life's Simple 7.

**Supplementary Table 5.**
The Hazard Ratios (HRs) of the associations between Life's Simple 7 adherence and risk of cardiometabolic diseases/death in BRHS participants aged 60-79 in 1998-2000, further adjusted for baseline prevalent cancer status (n=2662).

| LS7 Adherence | HR | 95% CI | p-value |
|---|---|---|---|
| Poor (Achieved 0-1 LS7 ideal levels) | Ref | - | < 0.01 |
| Intermediate (Achieved 2-3 LS7 ideal levels) | 0.73 | (0.64, 0.83) | |
| Ideal (Achieved 4+ LS7 ideal levels) | 0.58 | (0.50, 0.68) | |

Models used age as time scale, and adjusted for social class, alcohol intake, energy intake, and baseline prevalent cancer status. LS7 = Life's Simple 7.

**Supplementary Table 6.**
Cardiometabolic disease-free life expectancy and years of cardiometabolic disease-free life gained at age 60 for BRHS participants by Life's Simple 7 adherence (n=2662)

| LS7 Adherence | Cardiometabolic disease-free life expectancy at 60 y | | Years of cardiometabolic disease-free life gained at 60 y | |
|---|---|---|---|---|
| | Years | 95% CI | Years | 95% CI |
| Poor (Achieved 0-1 LS7 ideal levels, n = 403) | 18.42 | 16.93, 19.90 | Ref | - |
| Intermediate (Achieved 2-3 LS7 ideal levels, n = 1630) | 20.95 | 19.64, 22.25 | 2.53 | 1.44, 3.62 |
| Ideal (Achieved 4+ LS7 ideal levels, n = 629) | 22.79 | 21.27, 24.31 | 4.37 | 2.95, 5.79 |

Models used age as time scale, and adjusted for social class, alcohol intake, and energy intake. LS7, Life's Simple 7.

**Supplementary Table 7.**

Cardiometabolic disease-free life expectancy and years of cardiometabolic disease-free life gained at age 60 for BRHS participants by Life's Simple 7 adherence, stratified by social class.

| Manual workers (N = 1216) | Cardiometabolic disease-free life expectancy at 60 y | | Years of cardiometabolic disease-free life gained at 60 y | |
|---|---|---|---|---|
| LS7 Adherence | Years | 95% CI | Years | 95% CI |
| Poor (Achieved 0-1 LS7 ideal levels, n = 238) | 17.19 | 15.19, 19.20 | Ref | - |
| Intermediate (Achieved 2-3 LS7 ideal levels, n = 729) | 19.88 | 18.04, 21.71 | 2.68 | 1.18, 4.19 |
| Ideal (Achieved 4+ LS7 ideal levels, n = 229) | 21.65 | 19.39, 23.91 | 4.46 | 2.38, 6.53 |

| Non-manual workers (N = 1346) | Cardiometabolic disease-free life expectancy at 60 y | | Years of cardiometabolic disease-free life gained at 60 y | |
|---|---|---|---|---|
| LS7 Adherence | Years | 95% CI | Years | 95% CI |
| Poor (Achieved 0-1 LS7 ideal levels, n = 151) | 20.14 | 17.06, 23.23 | Ref | - |
| Intermediate (Achieved 2-3 LS7 ideal levels, n = 818) | 22.21 | 19.25, 25.18 | 2.06 | 0.30, 3.82 |
| Ideal (Achieved 4+ LS7 ideal levels, n = 377) | 24.26 | 21.17, 27.36 | 4.12 | 1.88, 6.35 |

Models used age as time scale and adjusted for alcohol intake and energy intake.
LS7, Life's Simple 7.

**Supplementary Table 8.**
Cardiometabolic disease-free life expectancy and years of cardiometabolic disease-free life gained at age 60 for BRHS participants by each Life's Simple 7 lifestyle metric (n=2662)

| LS7 lifestyle metric | Cardiometabolic disease-free life expectancy at 60 y | | Years of cardiometabolic disease-free life gained at 60 y | |
|---|---|---|---|---|
| | Years | 95% CI | Years | 95% CI |
| BMI | | | | |
| Poor (n = 365) | 18.82 | 17.29, 20.35 | Ref | - |
| Intermediate (n = 1417) | 21.28 | 19.95, 22.60 | 2.46 | 1.29, 3.63 |
| Ideal (n = 880) | 21.34 | 19.94, 22.74 | 2.53 | 1.28, 3.77 |
| | | | | |
| Blood Pressure | | | | |
| Poor (n = 1087) | 19.89 | 18.54, 21.23 | Ref | - |
| Intermediate (n = 1373) | 21.40 | 20.06, 22.74 | 1.52 | 0.69, 2.35 |
| Ideal (n = 202) | 22.98 | 20.97, 24.99 | 3.10 | 1.37, 4.82 |
| | | | | |
| Blood Glucose [a] | | | | |
| Intermediate (n = 1241) | 20.56 | 19.23, 21.89 | Ref | - |
| Ideal (n = 1421) | 21.23 | 19.90, 22.57 | 0.67 | -0.11, 1.45 |
| | | | | |
| Total Cholesterol | | | | |
| Poor (n = 1220) | 20.96 | 19.62, 22.30 | Ref | - |
| Intermediate (n = 967) | 21.00 | 19.61, 22.38 | 0.04 | -0.82, 0.89 |

| | | | | |
|---|---|---|---|---|
| Ideal (n = 475) | 20.65 | 19.12, 22.18 | -0.31 | -1.37, 0.76 |
| | | | | |
| Smoking | | | | |
| Poor (n = 346) | 17.73 | 16.25, 19.22 | Ref | - |
| Intermediate (n = 70) | 16.95 | 14.63, 19.27 | -0.78 | -2.98, 1.42 |
| Ideal (n = 2246) | 21.65 | 20.38, 22.92 | 3.92 | 2.74, 5.10 |
| | | | | |
| Physical Activity | | | | |
| Poor (n = 232) | 17.25 | 15.61, 18.88 | Ref | - |
| Intermediate (n = 1092) | 20.36 | 19.00, 21.71 | 3.11 | 1.75, 4.46 |
| Ideal (n = 1338) | 22.10 | 20.74, 23.45 | 4.84 | 3.37, 6.32 |
| | | | | |
| Elderly Diet Index | | | | |
| Poor (n = 1031) | 19.81 | 18.46, 21.16 | Ref | - |
| Intermediate (n = 991) | 21.30 | 19.90, 22.69 | 1.48 | 0.58, 2.39 |
| Ideal (n = 640) | 22.42 | 20.90, 23.93 | 2.61 | 1.48, 3.73 |

[a] Participants with poor glucose status were diabetic and excluded from the analysis.
Models used age as time scale, and adjusted for social class, alcohol intake, and energy intake.
BMI = body mass index; LS7 = Life's Simple 7.

**Supplementary Figure 1.**
Correlation matrix for Life's Simple 7 metrics

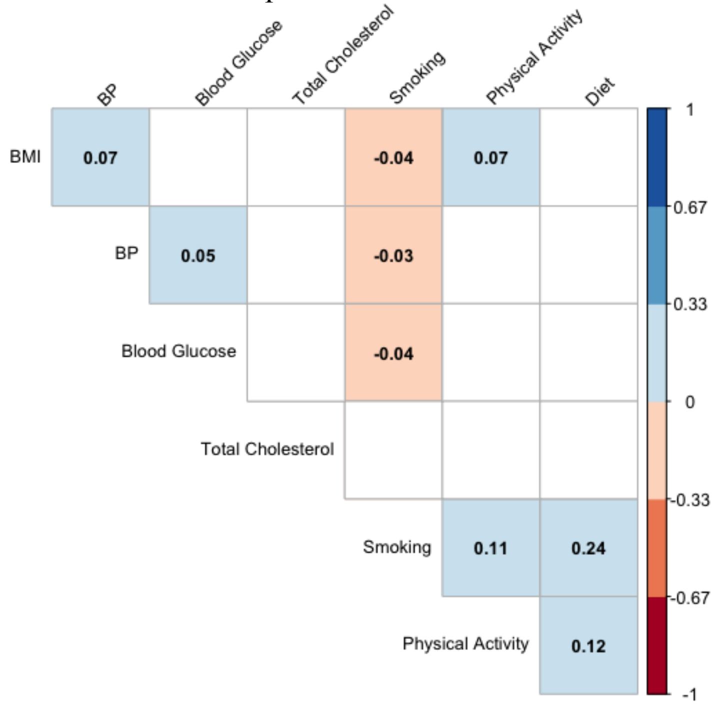


Spearman's rank correlation was used to measure the associations between each pair of LS7 metrics. Only significant correlation coefficients (p-value < 0.05) were kept in the figure.
BMI = body mass index; BP = blood pressure.

**Supplementary Figure 2.**
The Hazard Ratios and 95% Confidence Intervals of the associations between individual Life's Simple 7 metrics and risk of cardiometabolic diseases/death in BRHS participants aged 60-79 in 1998-2000 (n=2662).

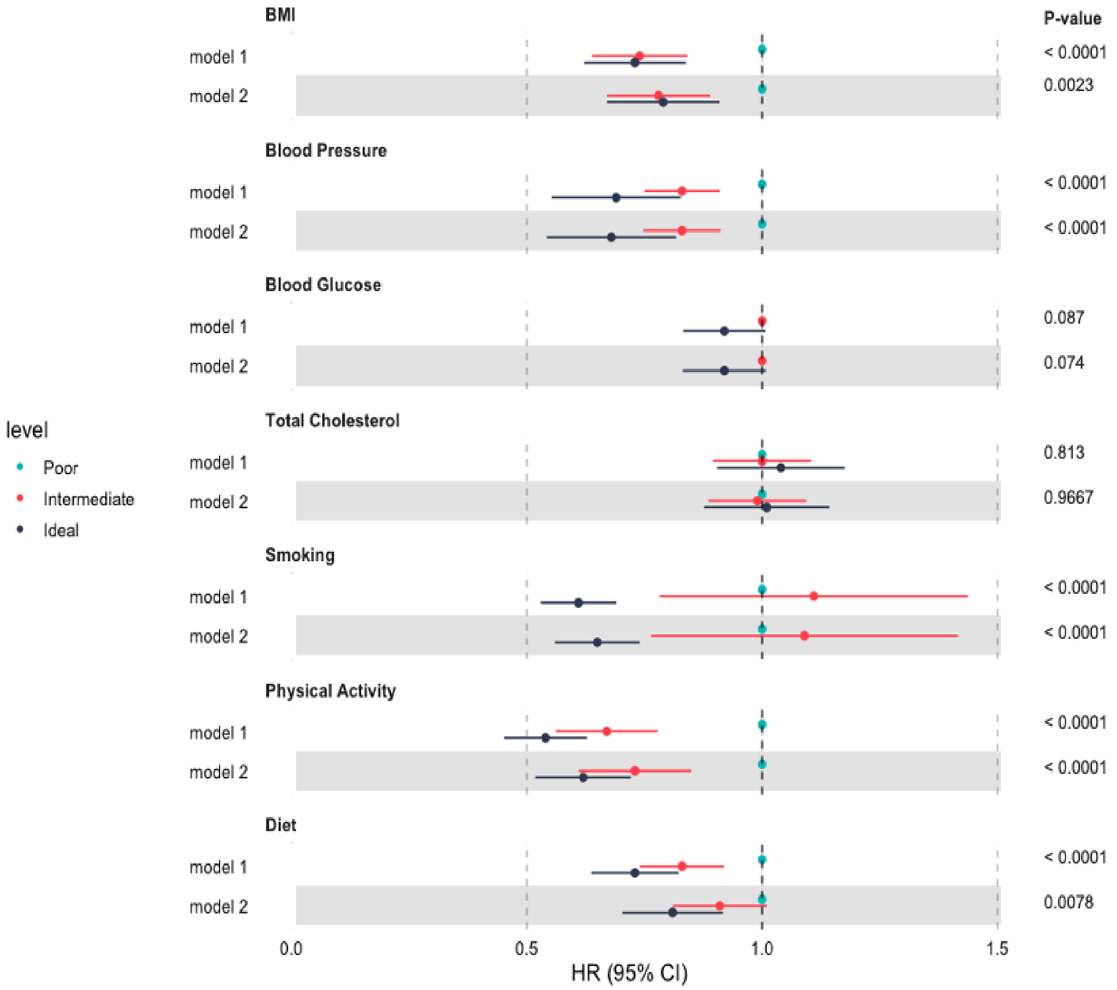


Model 1 used age as time scale and adjusted for social class, alcohol intake, and total energy intake. Model 2 used age as time scale and adjusted for social class, alcohol intake, total energy intake, and other LS7 factors.
BMI = body mass index.
