# Supplementary material for: Association of Life’s Simple 7 lifestyle metric with cardiometabolic disease-free life expectancy in older British men
Source: Commun Med (Lond). 2024 Jun 4;4:104. doi: 10.1038/s43856-024-00534-7 (PMC11150484; doi:10.1038/s43856-024-00534-7)
Supplement: Supplementary file 3 — Reporting Summary [file 43856_2024_534_MOESM3_ESM.pdf]

nature portfolio

Corresponding author(s): Qiaoye Wang

Last updated by author(s): May 15, 2024

Reporting Summary

Nature Portfolio wishes to improve the reproducibility of the work that we publish. This form provides structure for consistency and transparency in reporting. For further information on Nature Portfolio policies, see our Editorial Policies and the Editorial Policy Checklist.

Statistics

For all statistical analyses, confirm that the following items are present in the figure legend, table legend, main text, or Methods section.

n/a	Confirmed	
☐	☒	The exact sample size (*n*) for each experimental group/condition, given as a discrete number and unit of measurement
☐	☒	A statement on whether measurements were taken from distinct samples or whether the same sample was measured repeatedly
☐	☒	The statistical test(s) used AND whether they are one- or two-sided<br>*Only common tests should be described solely by name; describe more complex techniques in the Methods section.*
☐	☒	A description of all covariates tested
☐	☒	A description of any assumptions or corrections, such as tests of normality and adjustment for multiple comparisons
☐	☒	A full description of the statistical parameters including central tendency (e.g. means) or other basic estimates (e.g. regression coefficient) AND variation (e.g. standard deviation) or associated estimates of uncertainty (e.g. confidence intervals)
☐	☒	For null hypothesis testing, the test statistic (e.g. *F*, *t*, *r*) with confidence intervals, effect sizes, degrees of freedom and *P* value noted<br>*Give P values as exact values whenever suitable.*
☒	☐	For Bayesian analysis, information on the choice of priors and Markov chain Monte Carlo settings
☒	☐	For hierarchical and complex designs, identification of the appropriate level for tests and full reporting of outcomes
☒	☐	Estimates of effect sizes (e.g. Cohen's *d*, Pearson's *r*), indicating how they were calculated

Our web collection on statistics for biologists contains articles on many of the points above.

Software and code

Policy information about availability of computer code

Data collection	No software was used to collect the data.
Data analysis	STATA 17 statistical software (stpm2 command) was used. No novel code/software was developed.

For manuscripts utilizing custom algorithms or software that are central to the research but not yet described in published literature, software must be made available to editors and reviewers. We strongly encourage code deposition in a community repository (e.g. GitHub). See the Nature Portfolio guidelines for submitting code & software for further information.

Data

Policy information about availability of data

All manuscripts must include a data availability statement. This statement should provide the following information, where applicable:

- Accession codes, unique identifiers, or web links for publicly available datasets
- A description of any restrictions on data availability
- For clinical datasets or third party data, please ensure that the statement adheres to our policy

The raw data that support the findings of this study are not publicly available due to privacy and confidentiality considerations and are available from the BRHS study manager Lucy T. Lennon (l.lennon@ucl.ac.uk) upon reasonable request. Data are located in controlled access data storage at UCL. Source data for the figures can be found in Supplementary Table 6-8.

March 2021

Human research participants

Policy information about studies involving human research participants and Sex and Gender in Research.

Reporting on sex and gender	The British Regional Heart Study (BRHS) is a cohort of British male (biologically defined sex) participants. This current study included 2662 participants (men) from the BRHS.
Population characteristics	Of 2662 men being included in the analyses, the mean age at enrollment was 68.2 years. During a median follow-up of 19.3 years (Q1, Q3: 18.8, 19.9), 1764 (66.3%) participants developed cardiometabolic diseases or died.
Recruitment	Participants were randomly recruited from 24 primary care practices across Britain.
Ethics oversight	Ethical approval was provided by The National Research Ethics Service (NRES) Committee London–Central (Reference number: MREC/02/2/91).

Note that full information on the approval of the study protocol must also be provided in the manuscript.

Field-specific reporting

Please select the one below that is the best fit for your research. If you are not sure, read the appropriate sections before making your selection.

☐ Life sciences ☒ Behavioural & social sciences ☐ Ecological, evolutionary & environmental sciences

For a reference copy of the document with all sections, see nature.com/documents/nr-reporting-summary-flat.pdf

Behavioural & social sciences study design

All studies must disclose on these points even when the disclosure is negative.

Study description	A prospective cohort study with quantitative data.
Research sample	The research sample was drawn from the British Regional Heart Study (BRHS) 20-year follow-up data. The BRHS is a large ongoing cohort study that provides a geologically and socially representative cohort of UK males. The BRHS was started in 1978-1980, with 7735 middle-aged British men who were randomly selected from 24 general practices across the Great Britain. The 20-year follow-up data was collected from 1998-2000, which included sociodemographic, health, medication, lifestyle, and physical examination data. Mean age for the current research participants was 68.2 years.
Sampling strategy	Participants were randomly selected 24 general practices in 24 towns across the Great Britain.
Data collection	The BRHS 20-year follow-up data was collected through postal questionnaires and a lab-based physical examination. The postal questionnaires were self-completed by participants, and the physical measurements were carried out on each man by a team of specially trained nurses.
Timing	The BRHS 20-year follow-up data was collected in 1998-2000. Participants were followed up for their cardiometabolic disease and mortality until June 1st, 2018.
Data exclusions	4252 men who completed the 20-year follow-up questionnaires and underwent the physical examination were considered to be included in the current analysis. With pre-established analysis criteria, 1085 participants were excluded from the analysis due to prevalent myocardial infarction, stroke, or type 2 diabetes. 505 participants were excluded from the complete-case analysis due to missing lifestyle or sociodemographic information.
Non-participation	No participant dropped out during the current study follow-up.
Randomization	This is a prospective cohort study so no one is randomized to experimental group.

Reporting for specific materials, systems and methods

We require information from authors about some types of materials, experimental systems and methods used in many studies. Here, indicate whether each material, system or method listed is relevant to your study. If you are not sure if a list item applies to your research, read the appropriate section before selecting a response.

Materials & experimental systems

n/a	Involved in the study
☒	☐ Antibodies
☒	☐ Eukaryotic cell lines
☒	☐ Palaeontology and archaeology
☒	☐ Animals and other organisms
☒	☐ Clinical data
☒	☐ Dual use research of concern

Methods

n/a	Involved in the study
☒	☐ ChIP-seq
☒	☐ Flow cytometry
☒	☐ MRI-based neuroimaging
